# Oral selective estrogen receptor degraders and biomarker-driven therapy in ER-positive breast cancer: mechanisms, clinical evidence, and future directions

**DOI:** 10.3389/fonc.2026.1855542

**Published:** 2026-08-10

**Authors:** Anton Yu Alasheev, Raneem Hamama, Elena V. Petersen, Philipp Y. Maximov

**Affiliations:** Institute of Future Biophysics, Moscow Institute of Physics and Technology, Dolgoprudny, Russia

**Keywords:** biomarker-driven therapy, breast cancer, ctDNA, elacestrant, endocrine therapy, ESR1 mutations, estrogen receptor, fulvestrant

## Abstract

Breast cancer (BC) remains one of the most prevalent malignancies worldwide, with approximately 70% of cases being estrogen receptor-positive (ER+). Endocrine therapy targeting the estrogen receptor has revolutionized BC treatment, evolving from surgical oophorectomy to selective estrogen receptor modulators (SERMs) and aromatase inhibitors (AIs). Selective estrogen receptor degraders (SERDs) represent the latest advancement in hormonal therapy, offering complete receptor blockade and degradation. This review comprehensively examines the mechanisms of SERD action, their role in overcoming resistance to conventional endocrine therapy, and clinical applications of approved agents including fulvestrant, elacestrant, imlunestrant, and vepdegestrant, the first FDA-approved PROTAC estrogen receptor degrader. We discuss emerging oral SERDs such as giredestrant and camizestrant, novel PROTAC-based approaches, and combination strategies with CDK4/6 inhibitors, PI3K inhibitors, and other targeted agents. Special attention is given to ESR1 mutations as biomarkers for patient selection and therapy optimization. The review highlights key clinical trials including EMERALD, EMBER-3, SERENA-6, and lidERA that have shaped current treatment guidelines and points toward future directions in SERD development.

## 1 The relevance of breast cancer

### 1.1 Epidemiology

Breast cancer (BC) is one of the most pressing healthcare issues in terms of both incidence and mortality. According to 2022 statistics, BC ranks second among all cancer types by incidence for both sexes, accounting for 11.6% of new cases, second only to lung cancer (12.4%) (1). BC in women accounts for 23.8% of all oncological diseases, making it the most frequently diagnosed cancer among women worldwide (2). Breast cancer also ranks first in mortality among all cancer types in women, accounting for 15.4% of all cancer-related deaths in 2022 (2), underscoring the importance of early diagnosis and effective treatment methods.

Over the past two decades, the treatment of cancer has shifted from broadly cytotoxic chemotherapy toward molecularly targeted and biomarker-guided strategies that exploit the specific molecular dependencies of tumor cells (3). Small-molecule kinase inhibitors, inhibitors of hormone-biosynthetic enzymes, targeted protein degraders, and antibody-based agents now form the backbone of precision oncology across many tumor types (4, 5). Hormone receptor-positive breast cancer is a paradigmatic example of this evolution: the estrogen receptor (ER) is both a principal oncogenic driver and a highly tractable therapeutic target, and successive generations of endocrine agents have progressively refined how it is engaged. Within this landscape, selective estrogen receptor degraders (SERDs) represent the most recent advance, combining receptor antagonism with induced receptor degradation and increasingly guided by molecular biomarkers such as ESR1 mutations.

One of the key factors determining growth and malignant potential of many breast tumors is stimulation through the estrogen receptor (ER). Approximately 70% of all BC cases are ER-positive, meaning they contain estrogen receptors and are under hormonal stimulation (6). Moreover, the proportion of ER-positive BC continues to increase year by year (6). In this review, the authors examine achievements in the development of new selective ER degraders (SERDs) as one approach to hormonal therapy for BC.

## 2 The role of estrogen receptor in breast cancer pathogenesis

Estrogen receptor-α (ERα) is a nuclear transcription factor whose activation by estrogens leads to changes in gene expression controlling proliferation and survival of cells in various tissues, including breast (7). Estrogen binding to ER leads to receptor dimerization and binding to specific DNA sequences (estrogen response elements), initiating transcription of genes that stimulate the cell cycle and suppress apoptosis (8). Normally, this system regulates breast tissue growth and differentiation; however, in malignancy, it enables uncontrolled proliferation of tumor cells.

ERα consists of five domains, arranged from the N- to C-terminus of the protein (9).

These domains comprise an N-terminal region carrying the ligand-independent activation function-1 (AF-1), a central DNA-binding domain that anchors the receptor to estrogen response elements, a flexible hinge, a C-terminal ligand-binding domain (LBD) that houses the ligand-dependent activation function-2 (AF-2), and a short variable F region at the extreme C-terminus (9). Estrogen binding promotes receptor dimerization, recruitment of coactivators and transcription of proliferative genes, with maximal activity requiring cooperation between AF-1 and AF-2 (9). From a pharmacological standpoint the LBD is the decisive region, because it is where estrogens, antiestrogens and degraders bind and where the conformational switch that turns transcription on or off resides (10).

The LBD is folded into a bundle of about twelve α-helices that enclose a hydrophobic ligand-binding pocket, and its output is governed by the position of the C-terminal helix 12 (H12). When an agonist such as 17β-estradiol occupies the pocket, H12 packs against the body of the domain and completes the AF-2 surface – a hydrophobic groove that recruits the LXXLL motifs of p160 coactivators and switches transcription on (11). Antiestrogens bind the same pocket but carry a bulky side chain that prevents H12 from reaching this agonist position, so AF-2 remains non-functional (11). The ESR1 mutations that emerge under endocrine therapy, most frequently Y537S and D538G, act directly on this switch: they stabilize the agonist-like conformation of H12 in the absence of hormone, rendering the receptor constitutively active and resistant to estrogen deprivation (12).

**Figure 1:**
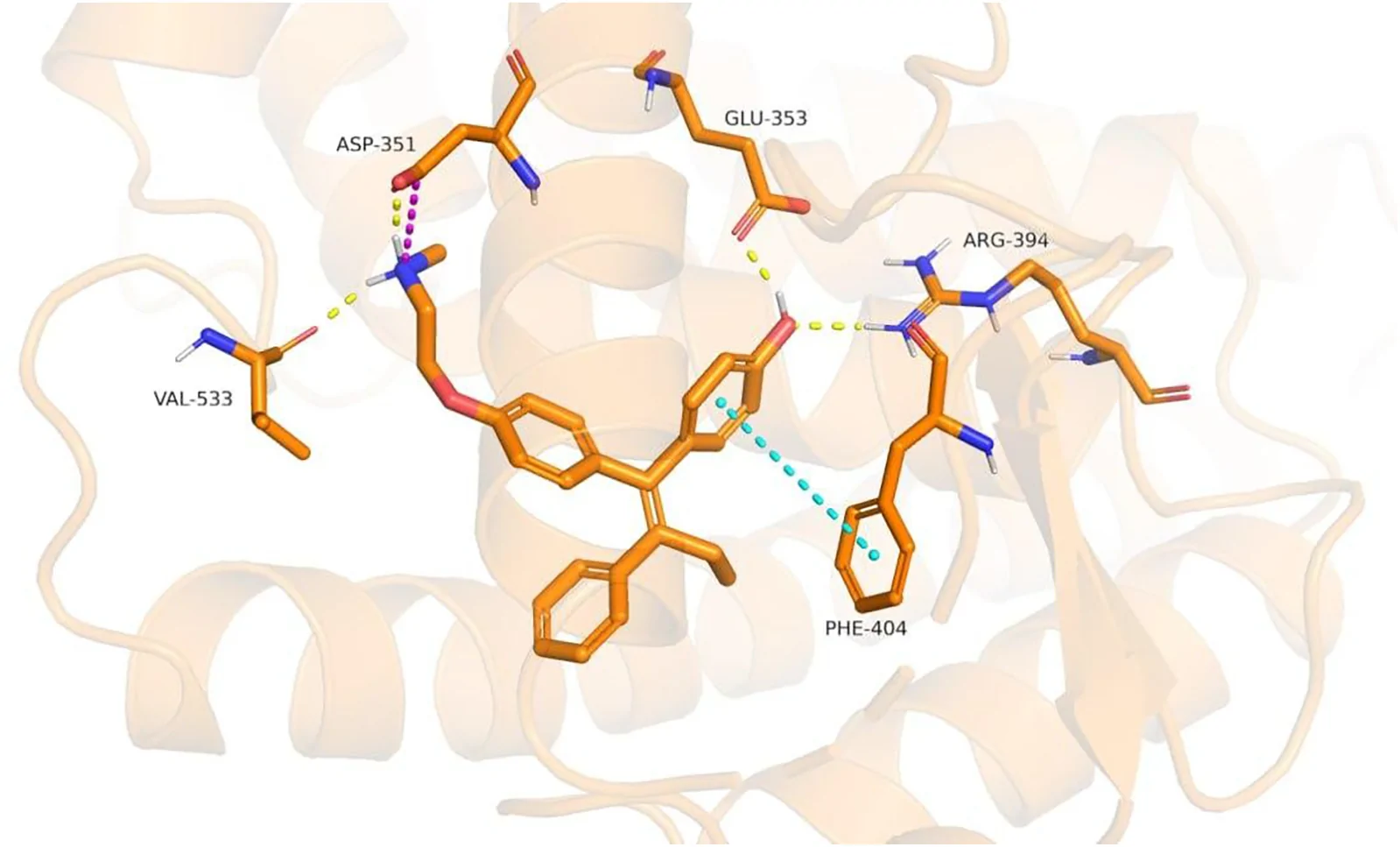
Estrogen receptor conformation bound to tamoxifen (SERM).

**Figure 2:**
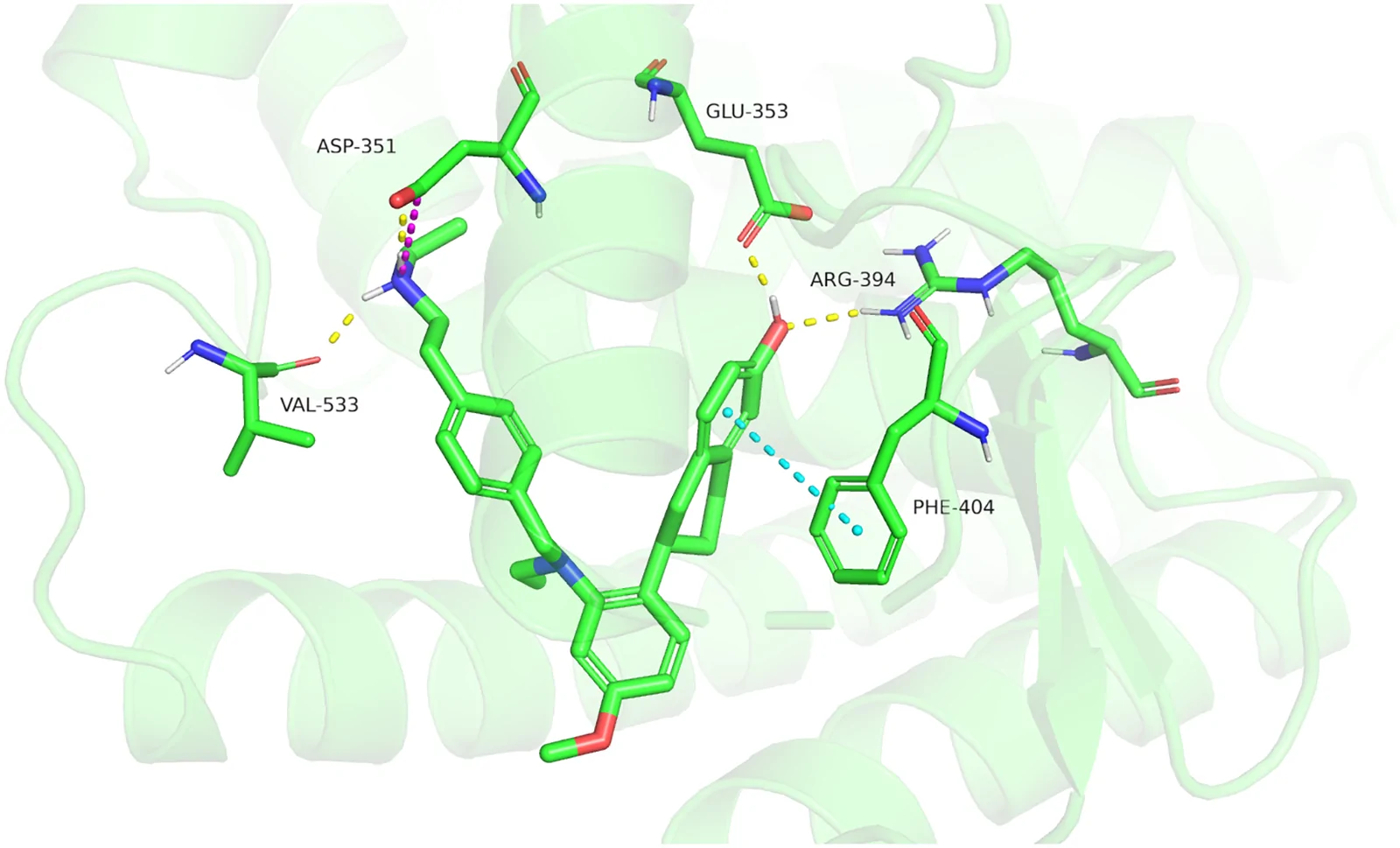
Estrogen receptor conformation bound to fulvestrant (SERD).

SERMs and SERDs both occupy this pocket but differ in their structural consequences. SERMs such as tamoxifen reposition H12 to block AF-2 while leaving the protein intact and AF-1 partially active; the receptor therefore persists in the cell and retains tissue-dependent agonist activity, which underlies the endometrial stimulation associated with tamoxifen (see **Figure 1**) (10). SERDs such as fulvestrant bind through a long, flexible side chain that not only prevents productive H12 positioning but also destabilizes the LBD fold and exposes hydrophobic surfaces that are normally buried; this abolishes both AF-1 and AF-2 activity, yielding a pure antagonist with no agonist effect, and simultaneously marks the receptor for ubiquitination and proteasomal degradation, lowering cellular ER levels (see **Figure 2**) (10). Because this degradation does not require the receptor to rest in an inactive conformation, SERDs remain effective against the constitutively active ESR1-mutant receptors that escape SERMs and aromatase inhibitors – providing the structural rationale for their use in endocrine-resistant disease (12).

The role of the ER in pathogenesis is confirmed by clinical observations: hormone-dependent tumors usually grow slower, more often metastasize to bones, and most importantly – are sensitive to hormonal function suppression (so-called endocrine therapy) (13). ER status is the most important prognostic and predictive factor in BC – it determines the molecular subtype of the tumor and largely predicts response to hormonal treatment (7). Estrogens effectively act as growth factors for ER-positive tumors, promoting invasion and disease progression (7). Therefore, blocking estrogen interaction with its receptor leads to suppression of such tumor growth – this is the basis of endocrine (hormonal) therapy. Overall, the estrogen receptor plays a central role in pathogenesis and progression of many BC cases, and targeting ER has become one of the main treatment strategies for hormone-sensitive BC.

## 3 History of BC treatment and evolution of hormonal therapy

Surgical treatment (mastectomy) was historically the first and for a long time the main method of breast cancer therapy. In the late 19th century, evidence emerged about the influence of ovarian hormones on tumor growth. In 1896, British physician George Beatson demonstrated that oophorectomy (ovary removal) in young women with advanced BC led to tumor regression (13). This became the first evidence of hormonal dependence in some BC cases and essentially represented an early form of endocrine therapy. Beatson wrote about the “influence of ovaries” stimulating tumor growth, and his work laid the foundation for using oophorectomy in patients with metastatic BC long before the discovery of drugs and the ER itself (13).

In the mid-20th century, other endocrine approaches were undertaken: for example, adrenalectomy and hypophysectomy (removal of adrenal glands or pituitary) to reduce estrogen levels in the body. These mutilating surgeries sometimes led to improvement in patients with metastatic BC but were accompanied by severe side effects (14). A breakthrough in understanding the mechanism of hormonal sensitivity came with the discovery in 1958 by Elwood Jensen of a specific receptor in breast tissue (13, 15). Jensen showed that the effect of hormone therapy occurs through interaction with the ER, thereby paving the way for developing targeted drugs against this receptor.

The era of drug hormonal therapy began in the second half of the 20th century. A turning point was the appearance of tamoxifen – a non-steroidal antiestrogen synthesized in the 1960s. Initially developed as a contraceptive, tamoxifen soon showed the ability to inhibit breast tumor cell growth (16). In the 1970s, the first successful clinical trials were conducted, and by the end of the decade, tamoxifen began to be used for metastatic BC treatment and subsequently in the adjuvant setting for ER-positive patients with non-metastatic BC. Tamoxifen belongs to the Selective Estrogen Receptor Modulator (SERM) class of drugs and acts as an antiestrogen in breast tissue, blocking estrogen stimulation of the tumor. Its introduction significantly improved treatment outcomes: the EBCTCG (Early Breast Cancer Trialists’ Collaborative Group) meta-analysis showed that 5 years of adjuvant tamoxifen therapy reduces annual BC mortality by approximately 31% and by 26.4% (relative) at 15 years after diagnosis compared to no hormonal therapy (17). This corresponds to a reduction in relative risk of death by nearly one-third and has led to a substantial increase in patient survival. According to some estimates, tamoxifen use has saved the lives of hundreds of thousands of women with BC over recent decades (16). Due to high efficacy, tamoxifen was included in the WHO list of essential medicines and became the “gold standard” of BC hormonal therapy in premenopausal patients, since it was discovered that postmenopausal women face risk of endometrial cancer (18).

The next step in the evolution of hormonal therapy was the development of drugs suppressing estrogen synthesis. In the 1990s, aromatase inhibitors (AIs) appeared – drugs blocking the aromatase enzyme and thereby preventing estradiol biosynthesis from androgens in peripheral tissues. These drugs are effective in postmenopausal women but do not work without suppression of ovarian function in premenopausal women (19). Major clinical studies showed that in postmenopausal women, modern aromatase inhibitors (letrozole, anastrozole, exemestane) exceed tamoxifen in reducing recurrence and death risk. According to accumulated data, in premenopausal women receiving ovarian function suppression, aromatase inhibitors versus tamoxifen reduced the relative risk of breast cancer recurrence by ~21% (19). In adjuvant therapy, AIs replaced tamoxifen in postmenopausal patients, with sequential regimens (e.g., 2–3 years tamoxifen + 2–3 years AI) also used (17). However, AIs suppress estrogen synthesis in all tissues, including bone. In women, this commonly leads to secondary osteoporosis; AIs may cause reduced bone density and bone fragility and can lead to osteoporosis (20).

In parallel, tamoxifen began to be used for breast cancer prevention in high-risk women. In 1998, the FDA approved tamoxifen for reducing invasive BC risk based on the NSABP-BCPT study (National Surgical Adjuvant Breast and Bowel Project – Breast Cancer Prevention Trial), in which a 5-year tamoxifen course reduced cancer risk by approximately 50% (21). Later, in 2007, based on results from the STAR trial (Study of Tamoxifen and Raloxifene), another drug – raloxifene – was approved for BC prevention in postmenopausal women with osteoporosis or increased cancer risk (21). However, raloxifene was not approved for adjuvant BC therapy due to lower efficacy than tamoxifen (22).

In the early 2000s, a new class of hormonal drugs appeared – selective estrogen receptor degraders (SERD), the first representative of which was fulvestrant. These drugs fundamentally differ from SERMs in that they not only block the receptor but also lead to its degradation. The appearance of fulvestrant marked another milestone in BC therapy, especially for overcoming resistance to tamoxifen and AI. In subsequent decades, treatment strategies for ER-positive cancer evolved toward combining hormonal and targeted drugs: for example, adding CDK4/6 inhibitors to endocrine therapy significantly improved progression-free survival (PFS) in metastatic BC (23). Nevertheless, endocrine therapy (whether SERM, AI, or SERD) remains the cornerstone of hormone-dependent breast cancer treatment. This review focuses on hormonal drugs – SERDs – their mechanisms of action, clinical use, and prospects.

## 4 Selective estrogen receptor degraders

### 4.1 Mechanism of SERD action

SERDs are drugs that bind to the LBD of the ERα but instead of activation, cause conformational changes in the receptor making it unstable. In particular, fulvestrant binding leads to changes in the spatial structure of the ERα and exposure of hydrophobic regions on the protein surface. These abnormal surface features are recognized by the cellular degradation system: ubiquitin ligase E3 is recruited to the receptor, marking ERα with ubiquitin, and the receptor is then degraded in proteasomes (24). Thus, SERDs not only block hormone binding to the receptor but also reduce ER levels in cells (receptor down-regulation) through complete protein degradation. Additionally, the “SERD-ER” complex does not function effectively: ER cannot translocate to the nucleus properly, which would be required for the activation of estrogen-responsive genes (25). The key difference between SERDs and SERMs is that SERMs (tamoxifen) are partial ER agonists. For example, in the endometrium, tamoxifen can partially act as a receptor stimulator, leading to undesirable side effects. Structurally, this is because tamoxifen blocks one activation function of the receptor (ligand-dependent AF-2) while the other function (AF-1) retains some activity (24). SERDs (fulvestrant and others) are “pure” ER antagonists: they block both activation functions (AF-1 and AF-2) and have no known agonistic properties (24). Fulvestrant, unlike tamoxifen, completely eliminates estrogen-dependent receptor activity and even suppresses its ligand-independent activity (e.g., mediated by phosphorylation) (24). The absence of agonist activity in SERDs means they do not stimulate the receptor even in estrogen-dependent tissues. In particular, they do not promote endometrial proliferation; fulvestrant does not increase endometrial cancer risk (unlike tamoxifen) (24). Thus, SERDs provide more complete and selective ER blockade without undesirable receptor stimulation.

An important feature of SERDs is the ability to overcome drug resistance arising during prolonged hormonal therapy with tamoxifen and AIs. One common resistance mechanism is mutation of the ESR1 gene encoding the ERα. Many of these mutations are localized in the ligand-binding domain and lead to constitutive receptor activation – meaning ER remains active even without estrogen (26). For example, substitution mutations Y537S and D538G stabilize the active conformation of the ERα and support expression of estrogen-dependent genes. Such tumors progress on aromatase inhibitors (reduced estrogen levels cannot inhibit the active ER) and are often resistant to SERMs (24). However, mutant receptors remain targets for SERDs: tumors with ESR1 mutations retain sensitivity to these drugs because SERDs cause ER degradation (24). Therefore, even when the mutant receptor is “locked” in an activated state, SERDs can eliminate it, preventing the tumor from using this ER-mediated growth pathway.

### 4.2 Role in clinical practice

The first and for a long time the only representative of the SERD class was fulvestrant. Fulvestrant is a steroid analog of estradiol with a long aliphatic side chain, developed by AstraZeneca (formerly ICI) and approved in 2002. It became the first SERD introduced into clinical practice (8).

Pharmacological features of fulvestrant: The drug is a highly lipophilic compound and is practically not absorbed orally. Therefore, it is produced as an injectable oil solution and administered intramuscularly. The standard regimen is 500 mg (two 5 ml injections) intramuscularly once monthly (with an additional dose at 2 weeks during the initial cycle). This route of administration creates some difficulties: it requires doctor visits for injections, is not as convenient as oral medications, and takes time to achieve maximum plasma concentrations (several months or more) (27). Nevertheless, fulvestrant has established its place in metastatic BC therapy. The drug is indicated when ER+ tumors progress on prior hormonal therapy – for example, at recurrence after tamoxifen or AIs. In clinical studies, fulvestrant demonstrated efficacy comparable to or exceeding standard hormonal drugs. In the FIRST study (Fulvestrant First-Line Study Comparing Endocrine Treatments), fulvestrant was non-inferior to anastrozole in first-line therapy of metastatic BC (28); in the CONFIRM study, higher-dose fulvestrant (500 mg vs 250 mg) improved PFS (HR = 0.80; 95% CI, 0.68–0.94; P = .006, corresponding to a 20% reduction in risk of progression) (29). In another major trial (FALCON), fulvestrant (500 mg) showed longer median PFS compared to anastrozole in patients without prior hormone therapy (16.6 vs 13.8 months) (30). These data confirm that fulvestrant is at least as effective as aromatase inhibitors in metastatic BC (8). In practice, fulvestrant is used as monotherapy in the second line and subsequently – in combination with targeted drugs. For example, the combination of fulvestrant with a CDK4/6 inhibitor (palbociclib, ribociclib, or abemaciclib) has become the standard treatment for many patients with advanced ER+HER2– BC, as it significantly increases PFS compared to hormonal therapy alone (23).

Side effects of fulvestrant are mainly associated with the estrogen-deficient state the drug achieves. Frequently observed are hot flashes, increased fatigue, arthralgia or myalgia (joint and muscle pain), and moderate nausea (27). These symptoms are similar to those occurring during menopause or with other endocrine drugs. Fulvestrant has no estrogen agonist activity, so it does not cause endometrial hyperplasia and does not increase thrombotic complication risk – this is its advantage over tamoxifen (29). Intramuscular injections can be accompanied by local reactions, though the drug is generally well tolerated. Fulvestrant is used in patients with liver disease as it does not cause severe systemic side effects.

For two decades, fulvestrant remained the only registered SERD drug. Limitations associated with its formulation and pharmacokinetics stimulated the search for new compounds with the possibility of oral use and increased activity. The goal in developing oral SERDs was to create drugs that would be convenient for patients, achieve high and sustained plasma concentrations, and more actively degrade ER, including mutant forms. The first representative of such drugs was elacestrant.

**Figure 3:**
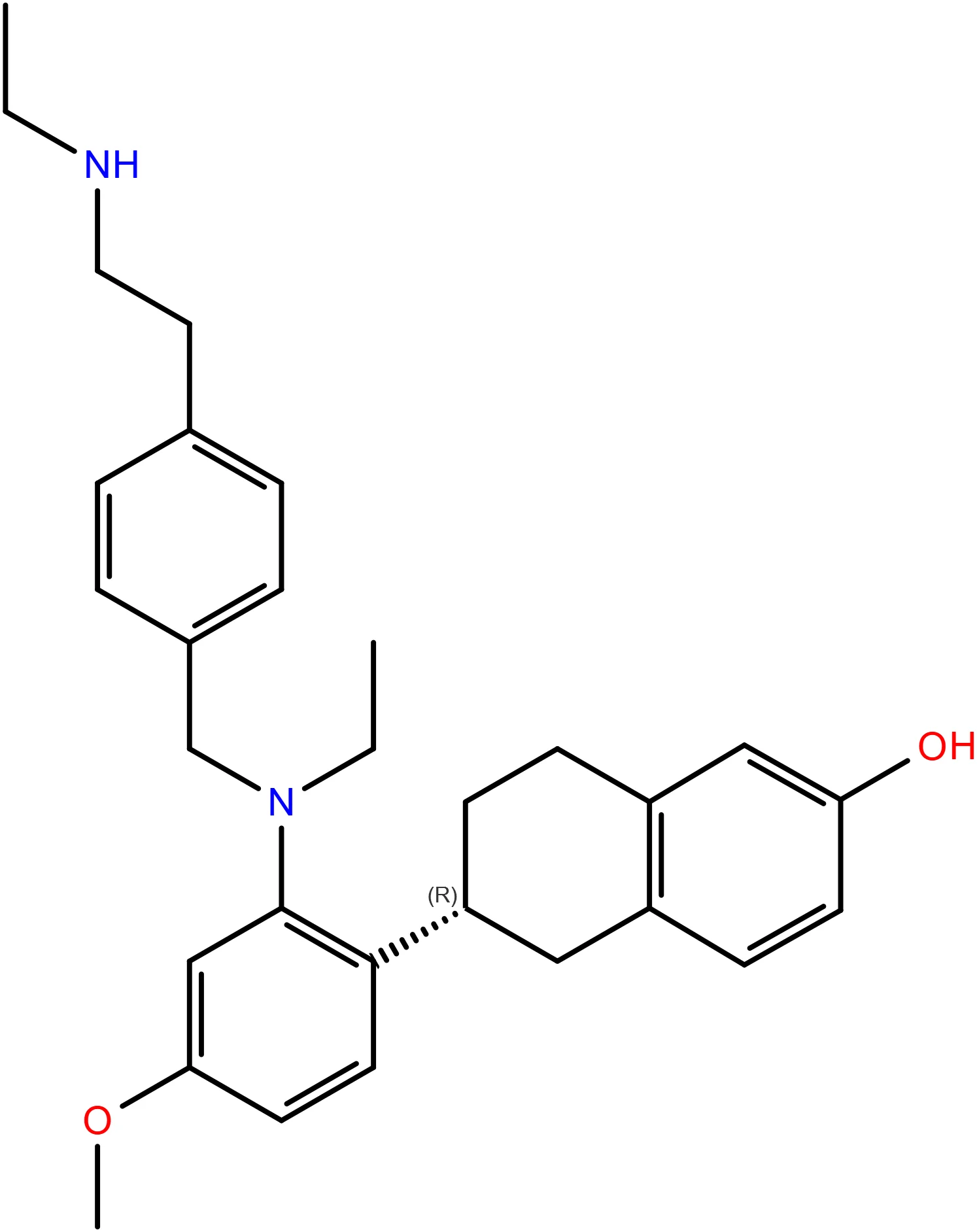
Chemical structure of elacestrant.

Elacestrant is a non-steroidal oral SERD developed by Radius Health (in partnership with Menarini). In January 2023, elacestrant became the first oral SERD to receive FDA approval in 20 years (31) (**Figure 3**). Indications: metastatic or advanced ER+HER2– breast cancer in postmenopausal women after at least one line of hormonal therapy, with confirmed ESR1 mutations (31). A companion diagnostic test (Guardant360 CDx) was also approved for detecting ESR1 mutations in tumors (31). This approval was based on results from the phase III EMERALD study, in which elacestrant was compared with standard endocrine therapy in patients with BC progression after AI + CDK4/6 inhibitor.

In the EMERALD study, patients (n=478) with metastatic ER+HER2– BC after 1–2 lines of endocrine therapy (previously receiving AI; >70% in combination with CDK4/6 inhibitor) were enrolled. Patients were randomized to elacestrant (345 mg orally daily) or physician’s choice of therapy (fulvestrant or other AI). About half the patients had ESR1 mutations detected at baseline (47% in both groups). The study showed a clinically significant improvement in progression-free survival (PFS) with elacestrant compared to standard hormonal therapy. The effect was particularly pronounced in patients with ESR1 mutations: median PFS was 3.8 months on elacestrant versus 1.9 months on standard therapy (fulvestrant or AI), representing a 45% reduction in progression risk (HR = 0.55; p=0.0005). In patients without ESR1 mutations, the difference in PFS was not significant (HR≈0.86, p>0.05) (31). Thus, elacestrant’s advantage is most evident in populations with ESR1-mutant tumors, which typically show resistance to aromatase inhibitors. Elacestrant demonstrated the ability to effectively act on mutant ER and inhibit growth of such tumors, hence its use is recommended specifically for patients with ESR1 mutations (31).

Regarding the safety profile of elacestrant compared to other endocrine drugs: in the EMERALD study, the most common adverse events were musculoskeletal pain (41% of patients on elacestrant vs 39% on control), nausea (35% vs 19%), and fatigue (26% vs 27%). Nausea was more frequent and distinguishing – occurring in most patients on elacestrant (usually grade 1-2). Grade 3–4 adverse events were rare; most commonly, elacestrant caused musculoskeletal pain (7% vs 1%). Laboratory abnormalities included elevated lipids and transaminases in some patients. Overall, like other hormonal drugs, elacestrant is generally well tolerated (32). Drug discontinuation due to side effects occurred in 12% of patients (approximately equal between groups). One of elacestrant’s advantages is its oral formulation – patients take a daily pill without the need for clinical visits, which increases convenience and compliance.

**Figure 4:**
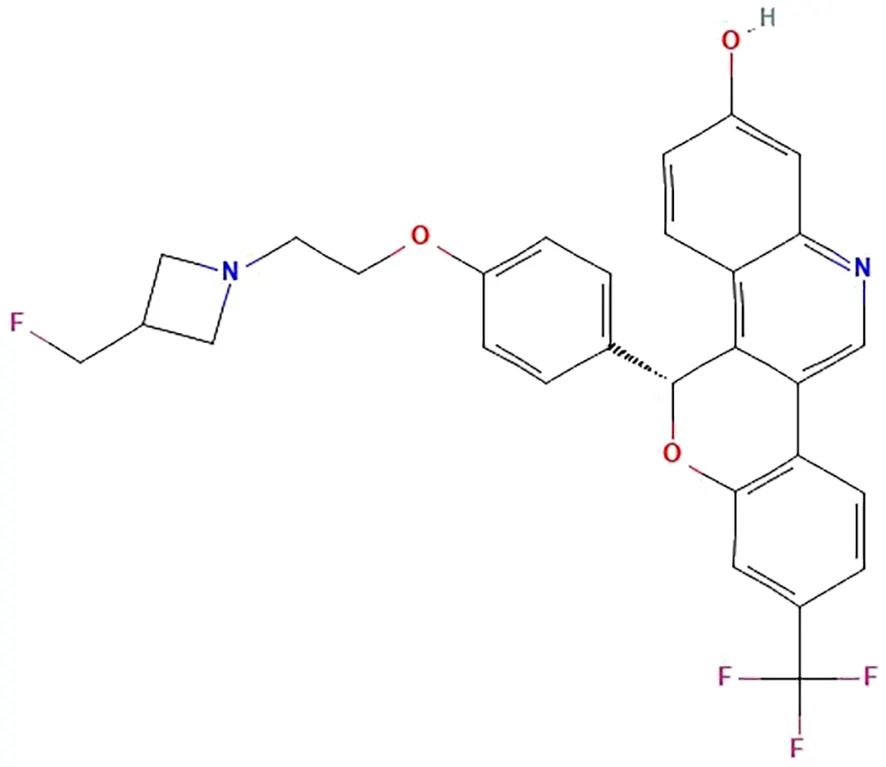
Chemical structure of imlunestrant.

Imlunestrant (Inluriyo, LY3484356) from Eli Lilly became another oral SERD approved by the FDA in September 2025 for treating adult patients with ER+, HER2−, ESR1-mutant locally advanced or metastatic BC after at least one line of endocrine therapy (33) (**Figure 4**). Imlunestrant represents an oral ER antagonist of the new generation with the ability to penetrate the blood-brain barrier, which is important for acting on brain metastases. Approval was based on results from the phase III EMBER-3 study, in which imlunestrant reduced the risk of progression or death by 38% compared to standard endocrine therapy in patients with ESR1 mutations (median PFS 5.5 versus 3.8 months; HR = 0.62; p=0.0008) (34). The objective response rate was 14.3% versus 7.7% in the control group. Of particular interest is the combination of imlunestrant with abemaciclib: median PFS was 10.9 months in the combination group versus 5.5 months with monotherapy (HR = 0.59; p<0.0001). Updated EMBER-3 data presented at SABCS 2025 showed median overall survival of 34.5 months in patients with ESR1 mutations compared to 23.1 months in the standard therapy group (HR = 0.60; p=0.0043) (35). Safety profile: neutropenia (30%), arthralgia (30%), nausea (26%), elevated AST (25%), fatigue (23%), diarrhea (22%). Serious adverse events occurred in 10% of patients. An important feature of imlunestrant is its ability to penetrate the CNS.

**Figure 5:**
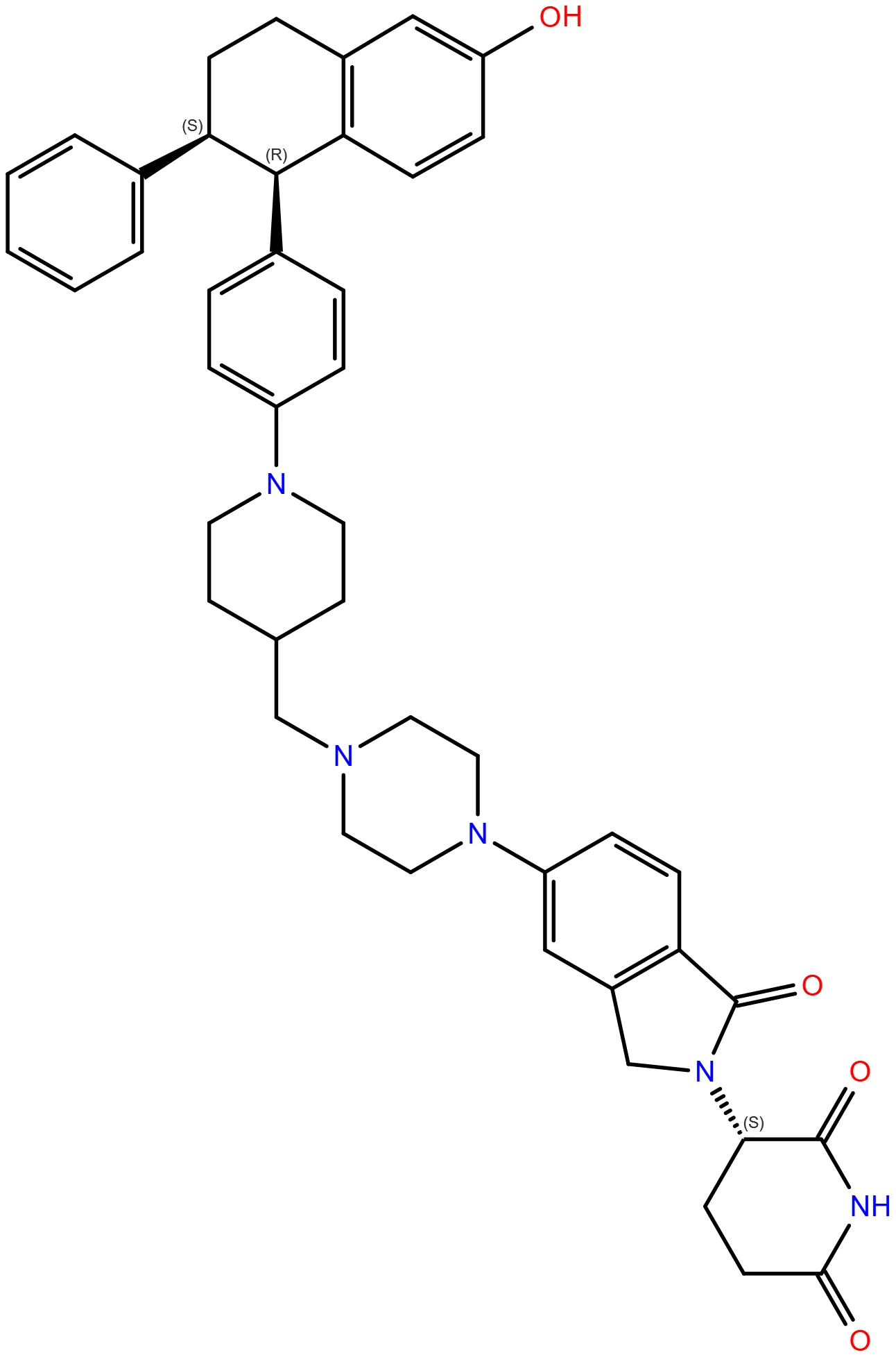
Chemical structure of vepdegestrant.

A fundamentally new approach involves molecules called PROTACs (proteolysis-targeting chimeras), which are bifunctional compounds that bind ER to an E3 ubiquitin ligase complex, leading to particularly effective receptor degradation (36). The leading drug is vepdegestrant (ARV-471) (**Figure 5**), developed by Arvinas and Pfizer: this oral PROTAC targeting ER has shown significant ER level reduction and antitumor activity in early studies, including in cases with ESR1 mutations (37). Updated phase 1b/2 VERITAC data of vepdegestrant in combination with palbociclib showed a median PFS of 13.9 months at the recommended phase 3 dose (RP3D) (200 mg) (including ESR1-mut subgroup). These phase 1b/2 results (38) were subsequently confirmed in the phase III VERITAC-2 trial, in which vepdegestrant was compared with fulvestrant in patients with ER-positive, HER2-negative advanced breast cancer progressing after CDK4/6 inhibitors and endocrine therapy. In the prespecified ESR1-mutated population (n=270) vepdegestrant significantly prolonged median progression-free survival (5.0 vs 2.1 months; HR 0.57; 95% CI 0.42–0.77; p=0.0001), with an objective response rate of 19% versus 4%; in the overall population the difference did not reach statistical significance (3.8 vs 3.6 months; HR 0.83; p=0.07), and overall survival data remain immature (39). On the basis of these data, vepdegestrant (VEPPANU) received FDA approval on 1 May 2026 for ER-positive, HER2-negative, ESR1-mutated advanced or metastatic breast cancer after at least one line of endocrine therapy (with an FDA-authorized ESR1 test), becoming the fourth approved ER degrader and the first PROTAC degrader approved in oncology (40, 41). It is administered at 200 mg orally once daily with food; the label includes warnings for QTc-interval prolongation and embryo-fetal

**Figure 6:**
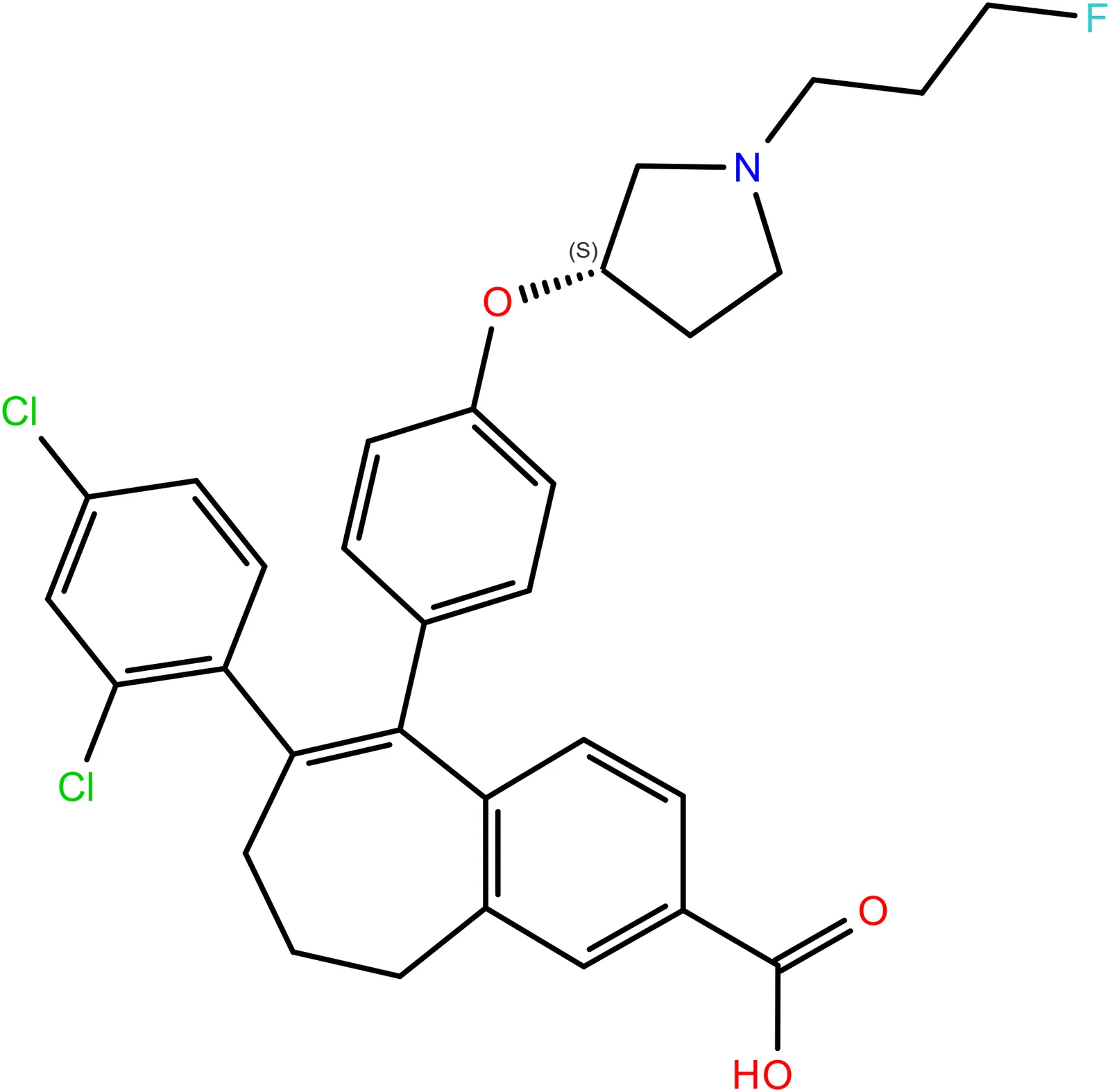
Chemical structure of amcenestrant.

Although elacestrant, imlunestrant and vepdegestrant share an indication – ESR1-mutated, ER+/HER2– disease after prior endocrine therapy – they are not interchangeable. Elacestrant was approved as monotherapy (EMERALD) with a modest absolute progression-free survival gain that was confined to the ESR1-mutant subgroup. Imlunestrant additionally showed a clinically meaningful benefit in combination with abemaciclib (EMBER-3, median PFS 10.9 vs 5.5 months) and penetrates the blood–brain barrier, making it attractive when a combination strategy or central nervous system involvement is a consideration. Vepdegestrant, the first PROTAC degrader, likewise produced benefit restricted to ESR1-mutant tumors (**Figure 6**). The failure of amcenestrant is instructive in this context: despite acceptable tolerability it yielded no progression-free survival advantage even in ESR1-mutant patients (AMEERA-3) and its phase III program was terminated, indicating that oral bioavailability alone is insufficient and that clinically effective SERDs must achieve potent binding and efficient degradation of both wild-type and mutant ER *in vivo*. At present, therefore, the oral SERDs are best regarded as biomarker-selected second-line options, with the choice between them driven by combination partner, central nervous system penetration and tolerability rather than by head-to-head efficacy data.

Four ER-targeted degraders are now in clinical practice: fulvestrant and the oral agents elacestrant and imlunestrant, together with the PROTAC degrader vepdegestrant for ESR1-mutated disease after prior endocrine therapy. Selected approved and late-stage agents are summarized in **Table 1** (approved) and **Table 2** (investigational and late-stage oral SERDs).

**Table 1:** Approved ER degraders.

Drug (route, dose)	Indication & status	Key efficacy	Main toxicities	Comments
Fulvestrant (IM, 500 mg monthly)	Metastatic ER+/HER2– BC; FDA approved 2002	FALCON: PFS 16.6 vs 13.8 mo vs anastrozole (HR 0.80)	Injection-site pain, hot flashes, fatigue, nausea	First approved SERD; parenteral only; slow onset
Elacestrant (oral, 345 mg daily)	ER+/HER2–, ESR1-mut advanced/metastatic BC after ≥1 line ET; FDA approved Jan 2023	EMERALD (ESR1-mut): PFS 3.8 vs 1.9 mo; HR 0.55 (0.39–0.77); p=0.0005	Nausea, fatigue, musculoskeletal pain (mostly grade 1–2)	First oral SERD; companion Dx Guardant360 CDx
Imlunestrant (oral, 400 mg daily)	ER+/HER2–, ESR1-mut advanced/metastatic BC after ≥1 line ET; FDA approved 25 Sep 2025	EMBER-3 (ESR1-mut, mono): PFS 5.5 vs 3.8 mo; HR 0.62; p=0.0008. +abemaciclib (ITT): PFS 10.9 vs 5.5 mo. OS 34.5 vs 23.1 mo (HR 0.60; p=0.0043)*	Fatigue, diarrhea, nausea (mostly grade 1–2)	CNS-penetrant; companion Dx Guardant360 CDx
Vepdegestrant (oral PROTAC, 200 mg once daily with food)	ER+/HER2–, ESR1-mut advanced/metastatic BC after ≥1 line ET (FDA-authorized ESR1 test); FDA approved 1 May 2026	VERITAC-2 (ESR1-mut): PFS 5.0 vs 2.1 mo; HR 0.57 (0.42–0.77); p=0.0001; ORR 19% vs 4%. OS immature	Fatigue, nausea; QTc prolongation; embryo-fetal toxicity	First PROTAC ER degrader approved in oncology

* OS interim — pre-specified significance boundary not met; data immature.

BC, breast cancer; ET, endocrine therapy; ESR1-mut, ESR1-mutated; IM, intramuscular; PFS, progression-free survival; OS, overall survival; ORR, objective response rate; HR, hazard ratio; ITT, intention-to-treat; CNS, central nervous system; Dx, companion diagnostic.

**Table 2:** Investigational and late-stage oral SERDs.

Drug (route, dose)	Indication & status	Key efficacy	Main toxicities	Comments
Giredestrant (oral, 30 mg daily)	Adjuvant ER+/HER2– early-stage (I–III) BC: NDA accepted, FDA priority review, PDUFA 30 Nov 2026. Giredestrant + everolimus for ESR1-mut advanced/metastatic BC: NDA under review, decision expected 18 Dec 2026. Not yet approved.	lidERA (adjuvant): 30% reduction in iDFS risk (HR 0.70). evERA (advanced, +everolimus): PFS benefit in ITT (HR 0.56) and ESR1-mut (HR 0.38; ~10 vs 5.4 mo).	Generally well tolerated; asymptomatic bradycardia	Potential first SERD in the adjuvant setting
Camizestrant (oral, 75 mg daily)	1L HR+/HER2– advanced BC with emergent ESR1 mutation; phase III, under regulatory review — not yet approved	SERENA-6 (ctDNA-guided switch, +CDK4/6i): PFS 16.0 vs 9.2 mo; HR 0.44. OS/PFS2 immature	Generally well tolerated; photopsia, sinus bradycardia	First ctDNA-guided switch strategy (Guardant360)
Amcenestrant (oral, SAR439859)	Phase III program discontinued (2022) — development stopped	AMEERA-3: no PFS benefit vs standard ET, including in ESR1-mut	Generally well tolerated	Oral bioavailability alone insufficient; illustrates need for potent degradation

BC, breast cancer; ET, endocrine therapy; ESR1-mut, ESR1-mutated; CDK4/6i, CDK4/6 inhibitor; PFS, progression-free survival; PFS2, second progression-free survival; OS, overall survival; iDFS, invasive disease-free survival; HR, hazard ratio; ITT, intention-to-treat; ctDNA, circulating tumor DNA; 1L, first line.

### 4.3 New SERDs in clinical studies

Following fulvestrant, active development of new oral SERDs began, aimed at exceeding fulvestrant’s efficacy. Beyond elacestrant, the most advanced drugs are amcenestrant, giredestrant, and camizestrant, briefly described below.

Λ Amcenestrant (SAR439859) from Sanofi completed several phase II-III studies in 2022. Unfortunately, in the randomized phase II trial (AMEERA-3), amcenestrant did not show superiority over standard hormonal therapy (fulvestrant or AI) in patients with metastatic BC after 1–2 lines of ET. The difference in median PFS was absent (3.6 vs 3.7 months), and even in patients with ESR1 mutations, PFS improvement was not statistically significant (42). Given amcenestrant’s acceptable safety profile, the phase III study (AMEERA-5, amcenestrant + palbociclib vs letrozole + palbociclib in first line) was discontinued early due to monotherapy inefficacy. The company terminated further amcenestrant development. This unsuccessful outcome demonstrates that not all SERDs are equally effective *in vivo* and that clinical efficacy requires strong binding and degradation of ER.

**Figure 7:**
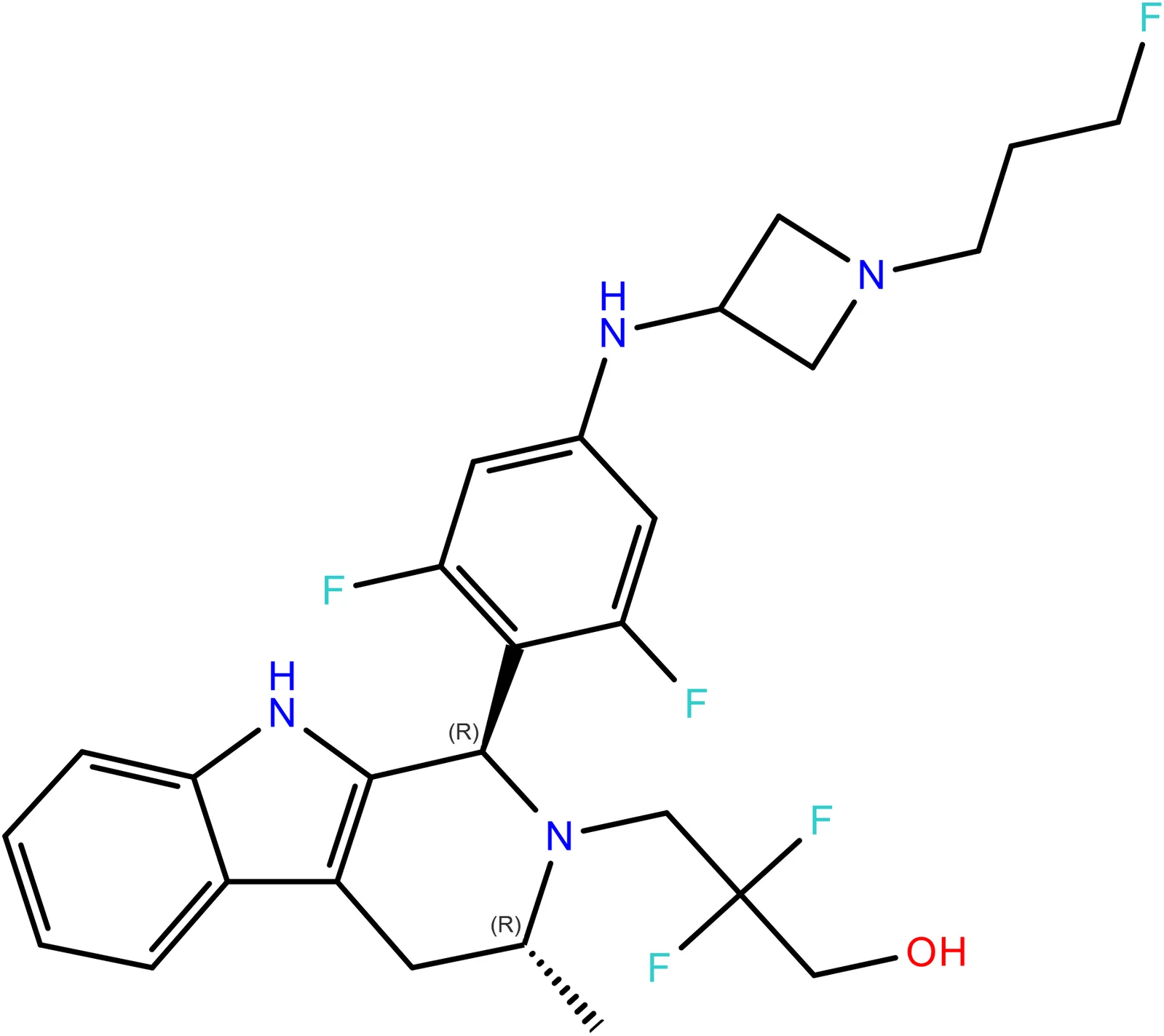
Chemical structure of giredestrant.

Λ Giredestrant (GDC-9545) from Roche demonstrated several important successes in 2025 (**Figure 7**). In metastatic BC after CDK4/6 inhibitors, positive results were seen in the phase III evERA study: the combination of giredestrant with everolimus improved PFS compared to standard endocrine therapy + everolimus (median PFS 8.8 versus 5.5 months; HR = 0.56; p<0.0001). In patients with ESR1 mutations, the effect was even more pronounced (HR = 0.38) (43) (44). Even more significant was the phase III lidERA study in adjuvant therapy: 3-year invasive disease-free survival (iDFS) with adjuvant giredestrant exceeded that with standard hormonal therapy (92.4% versus 89.6%; HR = 0.70; p=0.0014). This is the first improvement in adjuvant endocrine therapy results for ER+ BC in more than 20 years (45). However, giredestrant monotherapy in the phase II acelERA study did not show superiority over standard therapy in PFS in the overall population, although there was a signal of efficacy in subgroups with ESR1 mutations. Giredestrant is generally well tolerated with rare serious adverse events (46). In the neoadjuvant coopERA study, giredestrant with palbociclib exceeded letrozole + palbociclib in tumor proliferation reduction (measured by Ki-67 levels) (47). Results are awaited from the major phase III persevERA study (giredestrant + palbociclib vs letrozole + palbociclib in first line), expected in 2026 (43).

**Figure 8:**
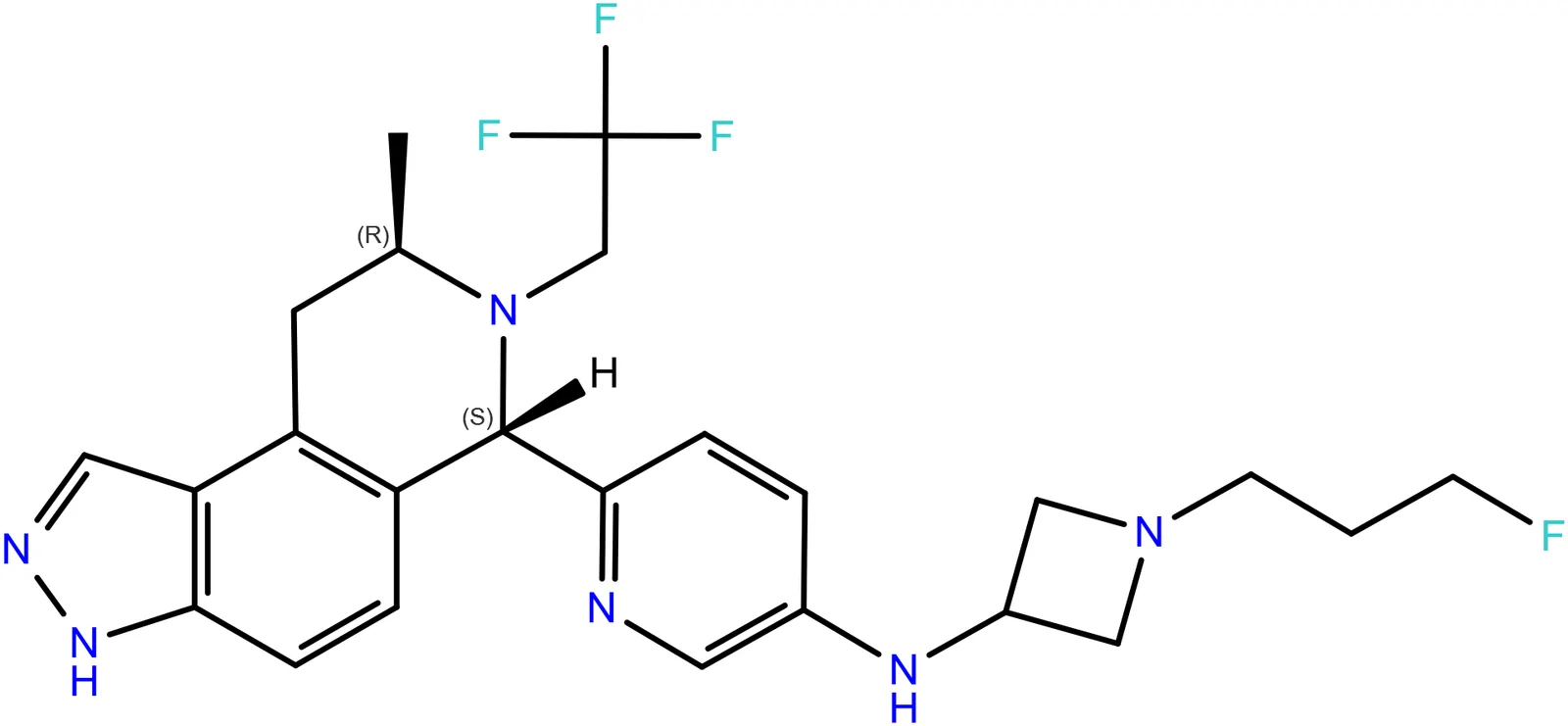
Chemical structure of camizestrant.

Λ Camizestrant (AZD9833) from AstraZeneca is one of the most promising next-generation oral SERDs (**Figure 8**). In the phase II SERENA-2 trial, two camizestrant doses (75 and 150 mg) were compared with fulvestrant in patients with metastatic BC who had previously received endocrine therapy. Both camizestrant doses showed superior PFS compared to fulvestrant: 7.2 months with camizestrant 75 mg, 7.7 months with camizestrant 150 mg, versus 3.7 months with fulvestrant (48). The benefit was observed across subgroups, including patients with ESR1 mutations (49). The next major step was results from phase III SERENA-6, presented at ASCO 2025 and published in NEJM (50, 51). In SERENA-6, an active surveillance strategy was used: in patients on first-line therapy (AI + CDK4/6 inhibitor), early detection of ESR1 mutations in circulating tumor DNA triggered early switch to camizestrant (in combination with the same CDK4/6i). This led to a 56% reduction in progression or death risk compared to continuing the same therapy (HR = 0.44; 95% CI 0.31-0.60). Median PFS with early switch to camizestrant + CDK4/6i was 16.0 months versus 9.2 months in the continuation group. The benefit was consistent across all CDK4/6 inhibitors (palbociclib, ribociclib, or abemaciclib) and all subgroups. These results are the first clinical evidence of monitoring ESR1 mutations for guiding therapy change before clinical progression (50, 51). Camizestrant is generally well tolerated; features included hot flashes and bradycardia in some patients, especially at higher doses. At 75 mg, such effects were less common (49). Camizestrant is not yet approved, but SERENA-6 data strongly support its use when resistance emerges. Results from the SERENA-4 study (camizestrant + palbociclib vs letrozole + palbociclib in first line) are awaited (51).

Covalent ER antagonists (e.g., H3B-6545) are also being studied, which irreversibly bind to specific mutant sites in the receptor, potentially overcoming resistance (52). An example is cSERD 29c, demonstrating antiproliferative activity superior to fulvestrant *in vitro* (53). Another compound (Compound 5) is being developed for treating patients with brain metastases, demonstrating favorable ADME properties *in vitro*, high oral bioavailability, and good CNS penetration (54).

Therapeutic combination of SERDs with other drugs is promising. Fulvestrant is already commonly used with CDK4/6 inhibitors, and new SERDs follow the same approach. In 2025, important data on combination SERD therapy emerged. The phase 2 ELEVATE study (a single-arm, open-label umbrella trial) demonstrated that adding elacestrant to targeted drugs yields clinically meaningful progression-free survival: the combination of elacestrant with everolimus (an mTOR inhibitor) achieved median PFS of 8.3 months (95% CI, 4.0–10.2), and with abemaciclib – 14.3 months (95% CI, 7.3–16.6) in previously treated patients, comparing favorably with historical data for endocrine therapy plus everolimus (mPFS 3.6–6.8 months) or CDK4/6 inhibitor switching (mPFS 5.3–9.4 months) (55). The combinations were well tolerated, confirming elacestrant’s potential in endocrine-based regimens for complete oral combinations. The phase III ADELA study has been launched to evaluate everolimus addition to elacestrant in patients with ESR1 mutations (56). The ELEGANT study has also started – comparing elacestrant with standard hormonal therapy in the adjuvant setting in patients with ER+ early BC and high recurrence risk (57). Clinical trials combining SERDs with PI3K/AKT/mTOR inhibitors are ongoing (58). The combination of giredestrant with everolimus in the evERA study may become the first complete oral regimen in the second line (43) (44). Of particular interest is combining SERDs with PROTAC degraders or other endocrine agents – sequential or combined action on ER through different mechanisms may overcome even cells that have developed resistance.

### 4.4 Mechanisms of endocrine resistance

Resistance to endocrine therapy is multifactorial, and SERDs address only part of it. The best-characterized mechanism is acquired mutation of ESR1 in the ligand-binding domain – most frequently Y537S and D538G – which locks the receptor in an active conformation, renders the tumor estrogen-independent and confers resistance to aromatase inhibitors; because SERDs degrade the receptor irrespective of its conformation, ESR1-mutant tumors generally retain sensitivity to them, which is the basis for the biomarker-defined indications of elacestrant, imlunestrant and vepdegestrant (26) (59).

Equally important, however, are ER-independent pathways that frequently drive progression in parallel. Hyperactivation of the PI3K/AKT/mTOR axis – most often through PIK3CA or AKT1 mutations or PTEN loss – sustains proliferation and survival despite receptor blockade and provides the rationale for combining endocrine therapy with PI3K inhibitors (alpelisib, inavolisib), the AKT inhibitor capivasertib or the mTOR inhibitor everolimus (58) (59). Reciprocal crosstalk between ER and receptor tyrosine kinase signaling, in particular the HER2/HER family and FGFR pathways, can bypass ER dependence and is associated with resistance to both SERMs and SERDs (60). Escape from CDK4/6 inhibition through dysregulation of the cyclin D–CDK4/6–Rb axis, and partial or complete loss of ER expression, represent further routes to refractory disease. Because these mechanisms often coexist, durable control increasingly depends on molecular profiling to pair each tumor with an appropriate combination partner rather than on endocrine monotherapy alone. In short, SERDs overcome ESR1-driven resistance but not the ER-independent escape pathways, so durable benefit increasingly depends on rational, molecularly profiled combinations rather than on endocrine therapy alone.

### 4.5 Clinical interpretation and limitations of oral SERD trials

These advances should nonetheless be interpreted with several caveats. The absolute progression-free survival gains in the pivotal trials are frequently measured in weeks to a few months – for example, 3.8 versus 1.9 months in EMERALD and 5.5 versus 3.8 months for imlunestrant monotherapy in EMBER-3 – and the statistically significant benefits are largely confined to ESR1-mutant subgroups, with neutral results in the overall populations of EMERALD and VERITAC-2. Overall survival data remain immature for most oral SERDs, so the long-term value of these progression-free survival improvements is not yet established. Cross-trial comparison is further limited by heterogeneous control arms, differing proportions of prior CDK4/6-inhibitor exposure and the absence of direct head-to-head studies. The SERENA-6 strategy of switching therapy upon emergence of ESR1 mutations in circulating tumor DNA is promising but raises questions of lead-time bias and optimal timing. Collectively, these considerations argue for cautious, biomarker-informed use of SERDs rather than uniform adoption across the ER-positive population.

## 5 Conclusion

Endocrine therapy has revolutionized breast cancer treatment, transforming a previously deadly disease into one that is controllable in many patients. The estrogen receptor drives growth in approximately 70% of breast tumors, and targeting ER has reduced BC mortality over recent decades. The evolution of hormonal drugs progressed from radical interventions (oophorectomy) to selective ER modulators (such as tamoxifen, which saved countless lives), and then to more complete and selective approaches. Selective ER degraders (SERDs) represent the latest step in hormonal therapy evolution. Fulvestrant, the first approved SERD, demonstrated that ER blockade and degradation can overcome resistance to tamoxifen and improve treatment outcomes (61). The appearance of new oral SERDs marks a new chapter in modern breast cancer therapy. Elacestrant became the first FDA-approved oral SERD in 20 years, improving outcomes in patients with tumors harboring ESR1 mutations. The year 2025 became a turning point for the SERD class. The SERENA-6 study demonstrated that monitoring ESR1 mutations in circulating DNA and early switching to camizestrant upon their detection reduces progression risk by 56% (50, 51). Giredestrant in the lidERA study became the first drug in more than 20 years to improve adjuvant endocrine therapy results for ER+ BC (45). In May 2026, vepdegestrant (VEPPANU) became the fourth approved ER degrader and the first PROTAC degrader approved in oncology, on the basis of the VERITAC-2 trial in ESR1-mutated disease (39, 41). Combinations of SERDs with targeted drugs (everolimus, CDK4/6 inhibitors, PI3K inhibitors) achieve high efficacy with good tolerability, paving the way for complete oral therapy regimens (43–55). Other drugs of this class (camizestrant, giredestrant, and others) are showing encouraging results in phase III clinical studies and are under regulatory review. These advances should nonetheless be read with measured optimism: the absolute progression-free survival gains are often modest and largely confined to ESR1-mutant tumors, overall-survival data remain immature, and no head-to-head trials yet establish the relative value of the individual oral degraders.

At the same time, the fight against hormone-dependent cancer is far from over. New challenges continue to emerge, such as development of resistance even to SERDs, activation of alternative tumor growth pathways, and the need for new therapeutic approaches. Biomarkers have become key: ESR1 mutations are now a major factor for SERD selection, and their monitoring in circulating DNA allows therapy changes before clinical progression (50, 59). Additionally, PIK3CA, HER2, FGFR1 mutations and other molecular alterations determine resistance patterns and guide combination partner selection with SERDs (59). The approach to ER+ BC is increasingly becoming comprehensive, combining endocrine drugs with targeted agents (kinase inhibitors, monoclonal antibodies) for maximum effect. The goal is to prolong endocrine sensitivity, delay chemotherapy, and improve the sequencing of biomarker-guided targeted combinations.

For physicians and researchers, it is important to monitor results of ongoing SERD clinical trials, study biomarkers of sensitivity, and develop new strategies for overcoming resistance. Selective ER degraders have secured their place in second-line therapy of metastatic breast cancer and may potentially be used in first-line and adjuvant settings, especially in combination with other drugs, allowing patients to achieve longer remissions. Incorporating SERDs into standard treatment may contribute to more individualized disease control across disease stages.
