# Correction: Oral selective estrogen receptor degraders and biomarker-driven therapy in ER-positive breast cancer: mechanisms, clinical evidence, and future directions

**DOI:** 10.3389/fonc.2026.1986541

**Published:** 2026-10-01

**Authors:** Anton Yu Alasheev, Raneem Hamama, Elena V. Petersen, Philipp Y. Maximov

**Affiliations:** Institute of Future Biophysics, Moscow Institute of Physics and Technology, Dolgoprudny, Russia

**Keywords:** biomarker-driven therapy, breast cancer, ctDNA, elacestrant, endocrine therapy, ESR1 mutations, estrogen receptor, fulvestrant

The funder “Ministry of Science and Higher Education of the Russian Federation, № 1024081900029-8(№ FSMG-2024-0049)” to PM was erroneously omitted.

The original version of this article has been updated.

